# Clozapine for the treatment of resistant psychosis – CLOZAPINE UNIT

**DOI:** 10.1192/j.eurpsy.2025.2102

**Published:** 2025-08-26

**Authors:** D. Tsaklakidou, M. Selakovic, D. Kosmidis, T. Pantazis

**Affiliations:** 1 Psychiatric Clinic, Sismanoglio General Hospital, Athens, Greece

## Abstract

**Introduction:**

Treatment resistant schizophrenia (TRS), according to HOWES et al. 2017, is defined as a patient’s condition in which, despite two or more treatment cycles with adequate dosage and duration of antipsychotic treatment, the patient’s condition does not improve to the extent expected with positive or negative or cognitive symptoms persisting. The heterogeneity of patients with resistant psychosis is high, and some may show the resistance in the course of the disease after years. However, several patients show resilience from the first psychotic episode.

**Objectives:**

Treatment resistant psychosis is probably a distinct subtype of schizophrenia, with a different etiopathogenetic mechanism. Clozapine is currently considered the drug of choice with proven efficacy, whereas atypical antipsychotic drugs are inferior in efficacy in the treatment of resistant psychosis.

**Methods:**

The mechanism of action of the drug is unknown and there is a potential for serious side effects to occur, which necessitates the adoption of a specific protocol for clozapine administration and patient monitoring in regular psychiatric clinical practice.

**Results:**

The Adult Psychiatric Clinic of Sismanoglio General Hospital in Athens, Greece, in its effort to create a systematic and integrated treatment and monitoring of patients who take clozapine, has created, in collaboration with the cardiology and hematology department of Sismanoglio Hospital a special unit for paients with treament resistant scizophrenia.

**Conclusions:**

The unit called “CLOZAPINE UNIT” will ensure the regular and continuous monitoring of patients receiving clozapine during and after their hospitalization in the psychiatric clinic.

**Disclosure of Interest:**

None Declared

